# Unsupervised Annotation Transfer in Phase-Contrast Microscopy Using a CycleGAN

**DOI:** 10.3390/s26154965

**Published:** 2026-08-05

**Authors:** Mokhaled N. A. Al-Hamadani, Stathis Hadjidemetriou, Gabor Szeman-Nagy, Paris A. Skourides, Andras Hajdu, Balázs Harangi

**Affiliations:** 1 Department of Data Science and Visualization, Faculty of Informatics, University of Debrecen, H-4032 Debrecen, Hungary; hajdu.andras@inf.unideb.hu (A.H.);; 2 Doctoral School of Informatics, University of Debrecen, H-4032 Debrecen, Hungary; 3 Department of Network and Computer Software Engineering, Polytechnic College–Hawija, Northern Technical University, Kirkuk 36001, Iraq; 4 Department of Information Technologies, University of Limassol, 3–5 Chaidariou Street, 3020 Limassol, Cyprus; stathis@uol.ac.cy; 5 Department of Microbial Biotechnology and Cell Biology, University of Debrecen, Life Sciences Building 1.102, 1 Egyetem Square, H-4032 Debrecen, Hungary; szeman-nagy.gabor@science.unideb.hu; 6 Department of Biological Sciences, University of Cyprus, 1678 Nicosia, Cyprus; skourip@ucy.ac.cy

**Keywords:** microscopy images, unsupervised learning, YOLO, domain adaptation, CycleGAN, annotation transfer

## Abstract

Domain shift between microscopy imaging domains poses a significant challenge for deploying deep learning-based cell detection models across different experimental setups. Manual annotation of new microscopy datasets remains resource-intensive and time-consuming. This study presents a detection-oriented Cycle-Consistent Generative Adversarial Network (CycleGAN)-based annotation transfer framework for adapting a labeled B16BL6 source domain to a HeLa target domain. Annotated B16BL6 melanoma microscopy images are translated into the visual appearance of HeLa microscopy data while retaining their original bounding-box annotations, enabling YOLOv8x detector training without full manual annotation of the target domain. Three YOLOv8x configurations were compared: a source-only bright B16BL6 baseline, an intensity-inverted dark B16BL6 baseline, and the proposed CycleGAN-translated B16BL6 → HeLa configuration. Performance was evaluated using standard object detection metrics on a manually annotated 100-frame HeLa target-domain subset, together with complementary unsupervised proxy metrics on the full unlabeled HeLa dataset. The CycleGAN-trained detector achieved the highest supervised target-domain performance, with a precision of 0.244, recall of 0.353, F1-score of 0.288, and mAP@0.50 of 0.186, compared with mAP@0.50 values of 0.027 and 0.009 for the bright and dark baselines, respectively. It also achieved the highest exploratory composite reliability score on the full HeLa sequence. These findings demonstrate that source-to-target image translation improves detector generalization under the investigated B16BL6 → HeLa phase-contrast microscopy domain shift, thereby reducing the need for extensive manual target-domain annotation.

## 1. Introduction

Phase-contrast time-lapse imaging is a crucial tool for detection and tracking cell division, supporting the investigation of cellular dynamics and the development of cancer therapies. Phase-contrast (PC) microscopy is label-free, non-invasive, and adequate for long-term observation of living cells. It is a well-established technique to monitor mitotic events in adherent cultures.

During mitosis, cells commonly round up, and their nuclei divide, forming halos of increased contrast in phase-contrast microscopy. In the final stage, cytokinesis produces daughter cells that temporarily retain these appearances. Manual detection of such events in time-lapse sequences is labor-intensive, time-consuming, and subjective, which suggests the need for automated and reliable analytical techniques.

Early approaches to automated mitosis detection were developed primarily for emission-based microscopy. Phase-imaging approaches relied on signal processing that often yielded blurry reconstructions [1]. Other studies correlated image features with ring-shaped patterns [2] or exploited symmetry in mitosis [3]. Markov chains [4] and conditional random fields (CRFs) [5,6] further improved detection by capturing temporal continuity.

With the rise of deep learning (DL), convolutional, recurrent, and attention-based architectures have achieved significant progress in microscopy image analysis. For instance, three-dimensional convolutional neural networks (3D CNNs) and hybrid convolutional neural network-long short-term memory (CNN–LSTM) models have been applied to extract spatiotemporal features for mitosis classification [7,8,9]. Complementary to detection, temporal tracking approaches reconstruct complete cell trajectories and division events using methods such as level sets [10], spatiotemporal association [11,12,13], and flow networks [14,15]. Attention-based detection and flow network tracking, however, remain computationally intensive [16].

Single-stage You Only Look Once (YOLO) detectors are becoming more popular in cellular and microscopic object detection because they perform localization and classification in a single inference pipeline [17]. In our previous work, we combined version 8 extra-large (YOLOv8x) with an enhanced Deep Simple Online and Realtime Tracking (DeepSORT) tracker to improve recall and temporal consistency for phase-contrast cellular analysis [18]. Beyond this application, attention-augmented YOLO variants have also been evaluated for blood-cell detection, demonstrating the suitability of the YOLO family for cellular localization [19]. However, these studies are mostly restricted to detection in a single domain and do not consider the performance degradation of a detector when transferred to different microscopy acquisition setups. Deep models remain sensitive to variations in illumination, image contrast, microscope settings, and imaging polarity [20].

From a theoretical perspective, domain adaptation considers the transfer of a model between source and target domains drawn from different distributions. The classical domain adaptation bound of Ben-David et al. [21] relates the error in the target domain to the error in the source domain, the discrepancy between the source and target distributions, and the error of a hypothesis that can work on both domains. This conceptual framework motivates techniques that reduce the gap between source and target while preserving relevant task information.

Unsupervised domain adaptation (UDA) for object detection includes several established methodological directions. Feature-alignment methods utilize adversarial learning to reduce discrepancies between source- and target-domain representations, as illustrated by Domain Adaptive Faster Region-Based Convolutional Neural Network (Faster R-CNN) [22]. Self-training approaches generate pseudo-labels for the target domain and iteratively refine the detector with confidence-filtered predictions [23]. Oza et al. [24] provide a broader overview of image-level translation, feature alignment, pseudo-label refinement, teacher–student learning, and hybrid methods. Such techniques can provide strong task-coupled adaptation but often require auxiliary discriminators, iterative predictions for the target domain, or detector-specific optimization.

Another view is image-level translation, which modifies the visual properties of the source or target domain before task-model training or inference. The Cycle-Consistent Generative Adversarial Network (CycleGAN) enables unpaired bidirectional image translation with adversarial, cycle-consistency, and identity-mapping constraints. In microscopy, Xing et al. [25] employed CycleGAN-based source-to-target translation together with iterative pseudo-label refinement for cross-modality cellular quantification across hematoxylin and eosin (H&E), immunohistochemistry (IHC), and phase-contrast images. Their study demonstrated the feasibility of transforming labeled microscopy images to an unlabeled target domain.

CycleGAN-based adaptation has also been investigated in other biomedical imaging contexts. Breen et al. [26] investigated the application of CycleGAN for cross-scanner mitosis detection in breast cancer histopathology and reported that its performance was dependent on the scanners and the detection frameworks. This observation is particularly relevant because cycle consistency encourages content preservation; it does not guarantee geometric invariance. Hence, the reuse of annotations after source-to-target translation should be empirically assessed rather than assumed to remain perfectly valid.

The present study does not introduce a new CycleGAN architecture, object detector, or general-purpose domain adaptation algorithm. Instead, it evaluates a detection-oriented combination of established components for annotation transfer in phase-contrast microscopy. These annotated B16BL6 source images are transformed to match the visual appearance of the HeLa target domain, and their existing Cell and Division bounding boxes are reused to train a fixed YOLOv8x detector. The detector is trained directly on translated source images that exhibit target-domain appearance, unlike target-to-source inference. Unlike pseudo-label self-training, the training annotations are derived from verified source-domain labels rather than from predictions generated on unlabeled target images.

Evaluation of unlabeled target-domain detections remains challenging as standard object detection metrics require ground-truth annotations. Indirect measures such as confidence, prediction agreement, pseudo-label stability, entropy, and detection-density metrics are commonly used in unsupervised adaptation research, but they are not substitutes for precision, recall, or average precision. They may suffer from model miscalibration, correlated errors, or systematic over-detection. Therefore, the proxy indicators used in this study are treated as exploratory diagnostics for the entire target sequence and are supported by standard detection metrics computed on an independently annotated HeLa subset.

Within this scope, the principal contributions of the study are:

1. **Detection-oriented annotation transfer pipeline:** An integration of unpaired source-to-target CycleGAN translation and YOLOv8x training for phase-contrast cell detection. The translated B16BL6 images approximate the HeLa visual appearance while reusing the existing Cell and Division bounding-box annotations without iterative target-domain pseudo-label generation.
2. **Empirical evaluation of annotation geometry:** A registration-focused evaluation that exploits scale-invariant feature transform (SIFT) correspondences, random sample consensus (RANSAC) affine estimation, keypoint displacement, and original-transformed bounding-box Intersection over Union (IoU) for spatial coherence between the original and translated source images. Unreliable registrations are reported separately rather than being treated as evidence of preservation.
3. **Direct target-domain ground-truth validation:** A small set of 100 HeLa frames that were carefully annotated by experts and independently verified, containing 726 cell and division instances, used exclusively for final evaluation. Precision, recall, F1-score, mean average precision at an IoU threshold of 0.50 (mAP@0.50), and mean average precision averaged across IoU thresholds from 0.50 to 0.95 (mAP@0.50:0.95) are reported without using the target annotations for training, checkpoint selection, and hyperparameter tuning.
4. **Controlled comparison of training-image configurations:** A comparison of bright B16BL6, dark B16BL6 with inverted intensity, and CycleGAN-transformed B16BL6 → HeLa training under the same YOLOv8x initialization, training duration, input resolution, and batch size. In this design, the detector configuration is held constant so that the principal experimental difference is the training-image condition.
5. **Complementary full-sequence proxy analysis:** An exploratory analysis of confidence, detection density, bounding-box area, inter-model consensus, and composite reliability over the complete HeLa sequence, together with a sensitivity analysis of different composite score weights. These are applied as complementary diagnostics rather than as substitutes for reliable detection metrics.

The study focuses on the B16BL6 → HeLa phase-contrast microscopy domain shift as a controlled source–target validation scenario. Both datasets share the same semantics for Cell and Division annotations but differ in cell morphology, illumination, contrast polarity, spatial resolution, and acquisition conditions. The framework is evaluated using standard object detection metrics on the annotated 100-frame HeLa subset, in the complete target sequence with complementary proxy metrics, and through a registration-based analysis of annotation geometry. The results are limited to this particular source–target pair, and the broader generalizability and relative effectiveness of the proposed method to other feature-alignment and pseudo-label-based UDA approaches remain to be established through additional controlled studies.

The remainder of this paper is organized as follows. Section 2 describes the datasets, problem formulation, CycleGAN architecture, reverse-mapping strategy, annotation geometry analysis, and YOLOv8x detection pipeline. Section 3 presents the experimental configuration and evaluates the proposed framework using ground-truth detection metrics, full-sequence proxy indicators, geometry preservation analysis, sensitivity analysis, and qualitative comparisons. Section 4 discusses the principal findings and limitations, and Section 5 concludes the study and outlines future research directions.

## 2. Materials and Methods

### 2.1. Datasets

#### 2.1.1. B16BL6 Source Dataset

The B16BL6 dataset was developed for cell detection and tracking and consists of 1800 grayscale phase-contrast microscopy frames with a spatial resolution of 1600×1200 pixels. The dataset was collected by the University of Debrecen using a continuous time-lapse near-infrared scanning microscopy system and contains melanoma-derived mouse cells in adherent culture. The dataset and its data-centric annotation pipeline are described in detail in [27], while the microscopy images and YOLO-format annotations are publicly available through the Open Science Framework (OSF) [28].

**Figure 1:**
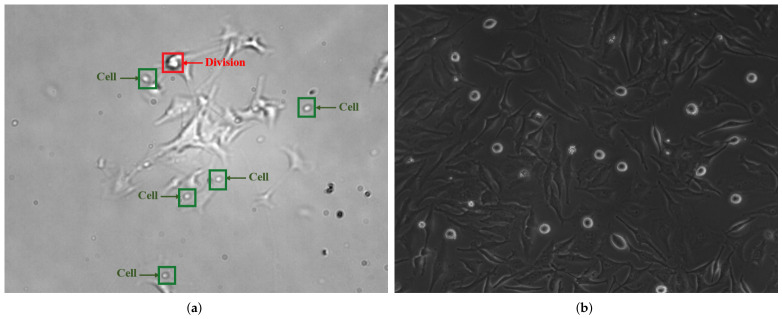
Sample microscopy frames used for domain adaptation: (**a**) B16BL6 source domain and (**b**) HeLa target domain.

The annotations comprise two operational classes: Cell, representing individual cells, and Division, representing cells undergoing mitotic division, as illustrated in Figure 1a. Of the 1800 frames, 342 frames (19.0%) were manually annotated and used to train the initial YOLOv8x detector. The detector was subsequently applied to the complete sequence to generate candidate annotations, which were verified by a biological expert and refined for spatial and temporal consistency using the previously developed fine-tuned YOLOv8x–Enhanced DeepSORT pipeline [18].

In the present study, the B16BL6 dataset serves as the labeled source domain $\left(X_{S},Y_{S}\right)$ for CycleGAN-based annotation transfer to the unlabeled HeLa target domain.

#### 2.1.2. HeLa Target Dataset

The target-domain dataset used in this study, referred to as HeLa, was provided by the University of Cyprus. This dataset was obtained from time-lapse microscopy recordings of HeLa cancer cell lines. The microscopy videos were converted into a sequence of image frames using ImageJ (version 1.54p) with the Bio-Formats Importer (Bio-Formats Plugins for ImageJ, version 8.1.1). The HeLa dataset comprises 2050 grayscale microscopy frames with a spatial size of 1300×1300 pixels, as shown in Figure 1b. The complete HeLa dataset was treated as unlabeled during CycleGAN training and detector adaptation. A subset of 100 frames was uniformly sampled across the complete 2050-frame time-lapse sequence to provide broad temporal coverage of the target dataset. The frame selection was performed independently of the outputs of the three evaluated detector configurations. The selected frames were annotated with bounding boxes for the Cell and Division classes by an expert annotator. A second expert subsequently reviewed all 100 frames to verify the accuracy of the bounding-box placement around the relevant cell locations and the correctness of the assigned class labels. Any identified inaccuracies were corrected before the ground-truth annotations were finalized. These annotations were used exclusively for final target-domain evaluation and were not used during CycleGAN training, YOLOv8x training, checkpoint selection, or hyperparameter optimization.

### 2.2. Problem Formulation

Let $X_{S}={\left\{\left(x_{i}^{S},y_{i}^{S}\right)\right\}}_{i=1}^{N_{S}}$ represent the labeled source-domain dataset, where $x_{i}^{S}\in R^{H\times W\times 3}$ denotes microscopy images and $y_{i}^{S}=\left\{\left(b_{j},c_{j}\right)\right\}$ indicates annotations comprising bounding boxes $b_{j}\in R^{4}$ and class labels $c_{j}\in \left\{1,\ldots ,K\right\}$. Let $X_{T}={\left\{x_{k}^{T}\right\}}_{k=1}^{N_{T}}$ represent the unlabeled target-domain dataset, which is characterized by varying visual statistics such as illumination, texture, and contrast, while depicting analogous biological structures.

The objective is to develop a detector $f_{\theta }:R^{H\times W\times 3}\to Y$ that generalizes well to $X_{T}$ utilizing annotations only from $X_{S}$. However, direct training on $X_{S}$ and testing on $X_{T}$ typically yields poor performance due to the domain shift between their marginal distributions: $p\left(x^{S}\right)\neq p\left(x^{T}\right),\;p\left(y^{S}|x^{S}\right)\approx p\left(y^{T}|x^{T}\right)$. Unsupervised domain adaptation seeks to address the distributional discrepancy by transferring the visual characteristics of $X_{T}$ to $X_{S}$ while maintaining annotation consistency.

### 2.3. CycleGAN Architecture

The CycleGAN approach is used to translate an image from a source domain to a target domain or perform image-to-image translation [29]. For the purpose of this study, CycleGAN is utilized to establish bidirectional mappings between the source and target domains. The framework is composed of:

- Generators: $G_{A}:X_{T}\to X_{S}$ (target-to-source) and $G_{B}:X_{S}\to X_{T}$ (source-to-target).
- Discriminators: $D_{S}$ distinguishes real source images $x^{S}$ from generated images $G_{A}\left(x^{T}\right)$, while $D_{T}$ distinguishes real target images $x^{T}$ from generated images $G_{B}\left(x^{S}\right)$.

Both generators utilize a residual network architecture with nine residual blocks (ResNet-9), instance normalization, and reflection padding. The discriminators follow the PatchGAN [30] design, classifying 70×70 overlapping image patches as real or fake.

The primary aim of CycleGAN comprises adversarial, cycle-consistency, and identity-mapping loss components. 

**Adversarial Loss.** The adversarial objective encourages each generator to produce images whose appearance is indistinguishable from that of the corresponding target domain. Utilizing the least-squares generative adversarial network (LSGAN) [31], the source-to-target adversarial loss is expressed as

(1) $$L_{\mathrm{GAN}}\left(G_{B},D_{T}\right)=E_{x^{T}}\left[{\left(D_{T}\left(x^{T}\right)-1\right)}^{2}\right]+E_{x^{S}}\left[D_{T}{\left(G_{B}\left(x^{S}\right)\right)}^{2}\right].$$

whereas the target-to-source adversarial loss is

(2) $$L_{\mathrm{GAN}}\left(G_{A},D_{S}\right)=E_{x^{S}}\left[{\left(D_{S}\left(x^{S}\right)-1\right)}^{2}\right]+E_{x^{T}}\left[D_{S}{\left(G_{A}\left(x^{T}\right)\right)}^{2}\right].$$

**Cycle-Consistency Loss.** Since paired source and target images are not available, cycle consistency is used to encourage the preservation of the essential image content during translation. An image translated to the opposite domain and subsequently mapped back should approximate the original input. The loss of cycle consistency can be written as

(3) $$L_{\mathrm{cyc}}=E_{x^{S}}\left[{∥G_{A}\left(G_{B}\left(x^{S}\right)\right)-x^{S}∥}_{1}\right]+E_{x^{T}}\left[{∥G_{B}\left(G_{A}\left(x^{T}\right)\right)-x^{T}∥}_{1}\right].$$

The cycle-consistency term helps to retain content and structure, but it does not guarantee complete geometric invariance mathematically.

**Identity-Mapping Loss.** An identity-mapping loss is used to prevent unnecessary modifications when an image already belongs to the output domain of a generator. In the source-to-target generator, the target-domain image should remain approximately unchanged, and the target-to-source generator should have an input that already belongs to the source domain. Thus, identity loss is defined as

(4) $$L_{\mathrm{id}}=E_{x^{T}}\left[{∥G_{B}\left(x^{T}\right)-x^{T}∥}_{1}\right]+E_{x^{S}}\left[{∥G_{A}\left(x^{S}\right)-x^{S}∥}_{1}\right].$$

This term complements cycle consistency by discouraging unnecessary appearance changes and supporting content preservation.

**CycleGAN Objective.** The full objective combines the bidirectional adversarial losses, cycle-consistency loss, and identity-mapping loss:

(5) $$L_{\mathrm{total}}=L_{\mathrm{GAN}}\left(G_{A},D_{S}\right)+L_{\mathrm{GAN}}\left(G_{B},D_{T}\right)+\lambda_{\mathrm{cyc}}L_{\mathrm{cyc}}+\lambda_{\mathrm{identity}}\lambda_{\mathrm{cyc}}L_{\mathrm{id}}.$$

Here, we set the cycle-consistency weight to $\lambda_{\mathrm{cyc}}=10$ following the standard CycleGAN formulation [29], while the identity-loss scaling factor was set to $\lambda_{\mathrm{identity}}=0.5$. Thus, the coefficient for the identity-mapping loss was 5. The identity term was used to prevent unnecessary modifications of images that already exhibit the appearance of the corresponding output domain but was not assumed to guarantee only photometric translation.

### 2.4. Reverse Mapping Strategy

**Conventional approach:** The conventional UDA approach translates the target images into the source style utilizing $G_{A}$ for inference, i.e., ${\tilde{x}}^{S}=G_{A}\left(x^{T}\right)$; subsequently, it applies the source-trained detector $f_{\theta }^{S}\left({\tilde{x}}^{S}\right)$.

**Our approach (reverse mapping):** We utilize $G_{B}$ to translate source images into the target style, hence generating synthetic training data:

(6) $${\tilde{x}}^{T}=G_{B}\left(x^{S}\right),\;{\tilde{X}}_{T}={\left\{\left({\tilde{x}}_{i}^{T},y_{i}^{S}\right)\right\}}_{i=1}^{N_{S}}.$$

The detector is subsequently trained directly on ${\tilde{X}}_{T}$ to acquire the target-domain appearance while utilizing existing annotations.

The following benefits accrue from utilizing the reverse approach instead of the traditional target-to-source translation approach. The suggested source-to-target reverse mapping upholds both the spatial relation and semantic significance of the associated annotation. The source annotations are therefore reused for the translated images under the assumption that the learned transformation introduces only limited spatial deformation. The correct results are obtained through the requirement of cycle consistency $\left(x^{S}\approx G_{A}\left(G_{B}\left(x^{S}\right)\right)\right)$ and identity criterion, whereupon $G_{B}$ is encouraged to modify target-domain appearance while retaining the underlying spatial organization of the source image.

**Figure 2:**
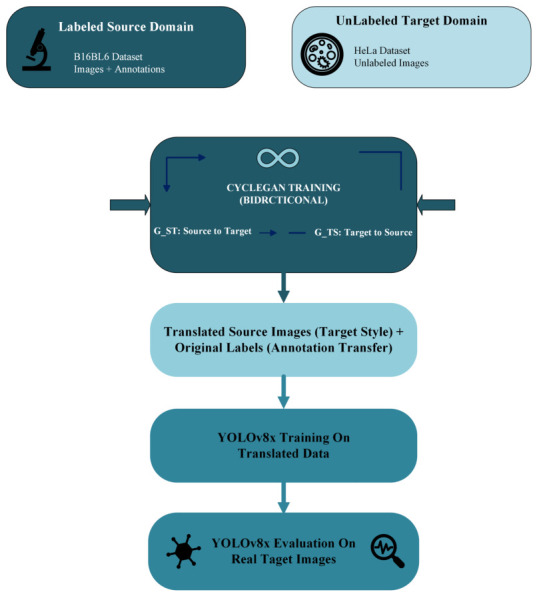
Flowchart of the proposed unsupervised domain adaptation pipeline based on a Cycle-Consistent Generative Adversarial Network (CycleGAN).

As shown in Figure 2, the process starts with the labeled source domain (B16BL6) and the unlabeled target domain (HeLa). CycleGAN is applied in a bidirectional training paradigm to establish mappings between the two domains, where the source images are translated into the target style appearance while reusing their original annotations under the evaluated spatial-correspondence assumption. Finally, the translated dataset is used to train the YOLOv8x detector, which is then assessed on real target-domain images.

This framework generates a translated source dataset ${\tilde{X}}_{T}$ containing images that approximate the target-domain appearance while reusing the corresponding source-domain annotations. Instead of generating labels from predictions in the target domain as in conventional pseudo-labeling, the labels are obtained from the annotated source images. Their spatial validity is evaluated through the registration-based annotation geometry analysis reported in Section 2.5 and Section 3.5.

### 2.5. Annotation Geometry Preservation Analysis

To quantitatively evaluate whether the CycleGAN translation introduced measurable spatial deformation that could compromise the validity of the transferred annotations, we performed an annotation geometry preservation analysis on paired original and translated B16BL6 images. Although the B16BL6 dataset contains 1800 annotated microscopy images, 1791 valid original–translated image pairs were available after CycleGAN inference and were used for the geometric analysis.

The SIFT features were extracted independently for each pair of images after contrast normalization. Candidate correspondences were filtered using Lowe’s ratio test, and RANSAC was applied to estimate a partial affine transformation from the original image to the translated image. A registration was considered valid if it contained at least eight filtered matches, at least six RANSAC inliers, and an inlier ratio of at least 0.50. Registrations with non-finite parameters, rotations larger than $45^{\circ }$, scales outside the range 0.5–1.5, or translations exceeding 50% of the image diagonal were considered invalid and excluded from the geometric estimates.

For valid registrations, geometric preservation was characterized using the median RANSAC inlier displacement, absolute translation magnitude, absolute rotation magnitude, and absolute scale deviation. These distributions were non-Gaussian and right-skewed; therefore, the results were summarized using the median, interquartile range (IQR), and tail percentiles rather than the mean and standard deviation.

We applied the estimated affine matrix to the four corners of each original YOLO bounding box to obtain the estimated transformation, which was directly correlated with annotation validity. Finally, the smallest axis-aligned rectangle of the transformed corners was compared with the original bounding box based on Intersection over Union (IoU) and center displacement metrics. The analysis assesses the spatial change implied by the estimated image-level transformation. Gradient-based structural similarity index measure (SSIM) and edge F1-score were retained only as supplementary structural indicators because they are also affected by the intended contrast, halo, and texture changes.

### 2.6. YOLOv8x Detection Pipeline

We utilized the YOLOv8x detection model due to its compelling tradeoff between accuracy and inference time [32]. YOLOv8x employs an anchor-free detection paradigm, an improved Feature Pyramid Network (FPN), and optimized loss functions. The YOLOv8x model exhibits the greatest size and performance of the YOLOv8 models, enabling enhanced representations of multi-scale features and superior object detection accuracy over a wider range of object sizes. This architecture was therefore chosen for use as the baseline detector in all the experiments carried out in this study.

## 3. Experiments and Results

The experiments were conducted using the B16BL6 and HeLa microscopy datasets. The training process was divided into three configurations: baseline (bright B16BL6), inverted (dark B16BL6), and proposed (CycleGAN(B16BL6 → HeLa)).

Evaluation was performed using two complementary protocols. Standard object detection metrics were calculated on the manually annotated 100-frame HeLa subset, whereas unsupervised proxy metrics were computed over the complete target-domain sequence. All the configurations were trained with YOLOv8x using the same initialization and training settings, as described in Section 3.1.

The entire experiment was executed on a cloud-based virtual machine equipped with a virtual 12-core CPU, 53 GB of system memory, an NVIDIA Tesla L4 GPU with 22.5 GB of memory, and 235 GB of allocated storage.

### 3.1. Training Configuration and Parameter Justification

The CycleGAN parameters used to generate the translated training dataset B16BL6 → HeLa are summarized in Table 1. Both generators employed a ResNet architecture with nine residual blocks and were paired with a 70×70 PatchGAN discriminator, instance normalization, reflection padding, and a least-squares adversarial objective. These settings follow the established CycleGAN formulation and were retained to provide a standard and reproducible image-translation configuration rather than introducing dataset-specific architectural modifications. The cycle-consistency weight was set to $\lambda_{\mathrm{cyc}}=10$, and the scale factor for the identity loss was set to $\lambda_{\mathrm{identity}}=0.5$. The identity term prevents unnecessary changes when an image already resembles the output domain and helps to preserve content, but it does not mathematically guarantee a purely photometric transformation.

Training used the Adam optimizer with a learning rate $2\times 10^{-4}$ and $\beta_{1}=0.5$. The learning rate was maintained for the first 100 epochs and then linearly decayed to zero over the following 100 epochs. During training, the images were resized to 512×512 pixels and randomly cropped to 256×256 pixels, allowing the model to learn local cellular appearance while maintaining a feasible computational requirement with a batch size of 4. Horizontal flipping was disabled to maintain a conservative geometry-preserving configuration. During translated-dataset generation, no resizing, cropping, or horizontal flipping was applied, thereby retaining the original image dimensions and annotation coordinate system. Based on visual inspection of a representative set of translated images, translation consistency, and absence of large structural artifacts, the epoch-122 checkpoint was selected before the target-domain ground-truth evaluation. This checkpoint was then fixed to generate the complete translated training dataset that was used in all the subsequent detector experiments.

The training configuration of YOLOv8x for bright B16BL6, dark B16BL6, and CycleGAN-translated B16BL6 → HeLa experiments is summarized in Table 2. All three detectors were initialized with the same COCO-pretrained YOLOv8x weights and trained separately for 100 epochs at an input resolution of 640 × 640 pixels and a batch size of 16. These parameters were explicitly specified and held constant in all the experiments, while the other optimization, regularization, augmentation, and validation settings were retained at the default values of Ultralytics YOLOv8x.

All the experiments were conducted using Python 3.12 and Ultralytics version 8.4.108. A fixed random seed of 42 was applied throughout the experimental pipeline to improve reproducibility.

To isolate the influence of the training-image condition, we implemented this matched configuration. Thus, the three detector experiments differed only by the type of training images used, either the original bright B16BL6 images, the inverted dark B16BL6 images, or the CycleGAN-translated B16BL6 images. No detector hyperparameter was selected or adjusted using the manually annotated HeLa evaluation subset, thereby avoiding target-domain hyperparameter tuning and ensuring a controlled comparison.

### 3.2. Evaluation Metrics

Two complementary evaluation protocols were employed. First, standard object detection metrics, including precision, recall, F1-score, mAP@0.50, and mAP0.50:0.95, were computed on the manually labeled 100-frame HeLa subset. Second, unsupervised proxy metrics were calculated across the entire HeLa sequence to characterize large-scale target-domain detection behavior beyond the annotated subset.

Therefore, to evaluate the effectiveness of the three configurations on the HeLa microscopy dataset, we employed the following unsupervised proxy metrics to compare their relative detection performance:

1. Detection Count per Image ($N_{\det}$): This metric computes the average number of detections.
2. Confidence Score (*C*): It measures the model’s certainty in its predictions.
3. Bounding-Box Area Distribution ($A_{\mathrm{bbox}}$): It quantifies and evaluates the dimensional parameters and uniformity of the anticipated bounding boxes.
4. Inter-Model Consensus Rate ($R_{\mathrm{cons}}$): This metric describes the proportion of detections from one configuration that spatially overlap, at IoU ≥0.50, with a class-consistent detection from at least one other configuration. It measures cross-model spatial agreement only and should not be interpreted as detection accuracy, pseudo-precision, or evidence of correctness because different models may exhibit correlated errors or agree on the same false detections.
5. Composite Score ($S_{\mathrm{composite}}$): We derive an aggregate performance indicator by linearly combining normalized measurements according to empirically determined weights reflecting their diagnostic significance. The normalized terms are adjusted in relation to their maximum values to facilitate comparability while minimizing susceptibility to outliers.
  (7) $$S_{\mathrm{composite}}^{\left(m\right)}=0.5\;C_{\mathrm{norm}}^{\left(m\right)}+0.3\;R_{\mathrm{cons},\mathrm{norm}}^{\left(m\right)}+0.2\;N_{\mathrm{inv},\mathrm{norm}}^{\left(m\right)}.$$
  where *m* denotes a detector configuration, $C_{\mathrm{norm}}^{\left(m\right)}$ is the normalized mean confidence, $R_{\mathrm{cons},\mathrm{norm}}^{\left(m\right)}$ is the normalized inter-model consensus rate, and $N_{\mathrm{inv},\mathrm{norm}}^{\left(m\right)}$ is the normalized inverse detection density. These terms are defined as
  (8) $$C_{\mathrm{norm}}^{\left(m\right)}=\frac{{\bar{C}}^{\left(m\right)}}{\max_{j}{\bar{C}}^{\left(j\right)}},\;R_{\mathrm{cons},\mathrm{norm}}^{\left(m\right)}=\frac{R_{\mathrm{cons}}^{\left(m\right)}}{\max_{j}R_{\mathrm{cons}}^{\left(j\right)}},$$
  and
  (9) $$N_{\mathrm{inv},\mathrm{norm}}^{\left(m\right)}=\frac{1/{\bar{N}}_{\det}^{\left(m\right)}}{\max_{j}\left(1/{\bar{N}}_{\det}^{\left(j\right)}\right)}.$$

### 3.3. Comparative Evaluation of Training-Image Configurations

To assess the individual contributions of each component in our proposed framework, we evaluate three distinct configurations for the (B16BL6 → HeLa) domain adaptation task. All three configurations used the same YOLOv8x model to facilitate a fair comparison and were trained for 100 epochs, as mentioned in Table 2.

1. Baseline (bright B16BL6):
  We trained YOLOv8x on the original 1800 bright B16BL6-annotated frames. This is the standard transfer learning scenario: the model is trained on the source domain and directly applied to the target domain without adaptation. Therefore, the performance illustrates degradation caused by a domain shift, where this model over-detected, averaging 85.7 detections per image and low confidence (0.44).
2. Inverted contrast-polarity baseline (dark B16BL6):
  To examine whether matching the broad contrast polarity of the HeLa target images was sufficient to reduce the source–target discrepancy, a controlled dark B16BL6 baseline was generated using direct pixel-wise intensity inversion, defined for the 8-bit source images as $I_{\mathrm{dark}}=255-I$. No histogram equalization, contrast normalization, or geometric transformation was applied. The original B16BL6 annotations were retained, and the grayscale images were replicated across three channels for YOLOv8x training.
  This configuration was included only as a contrast-polarity control and not as a complete domain adaptation method. The supervised evaluation presented in Section 3.4 show that intensity inversion alone did not improve target-domain detection performance and produced lower precision, recall, F1-score, and mAP values than the original bright B16BL6 baseline.
3. Proposed (CycleGAN (B16BL6 → HeLa)):
  Finally, we employed our proposed framework by training a CycleGAN to learn a bidirectional mapping between the B16BL6 and HeLa domains, as illustrated in Section 3. The epoch-122 CycleGAN checkpoint described in Section 3.1 was used to translate the annotated B16BL6 images into the HeLa visual style. This approach created a synthetic dataset ${\tilde{X}}_{T}={\left\{\left(G_{B}\left(x_{i}^{S}\right),y_{i}^{S}\right)\right\}}_{i=1}^{N_{S}}$ that maintains original annotations while matching target appearance. Then, YOLOv8x was trained on this CycleGAN-translated dataset using the same concept. Representative target-domain detection outputs from all three configurations are presented later in Section 3.7.

Table 3 summarizes the full-sequence proxy analysis for the three training configurations. The CycleGAN-translated B16BL6 → HeLa configuration produced a mean detection density of 46.4 detections per image and the highest mean confidence of 0.69. It also obtained the highest exploratory composite score (0.933), compared with 0.726 for the bright B16BL6 baseline and 0.786 for the dark B16BL6 baseline.

The indicators in Table 3 should be interpreted as descriptive measures of full-sequence prediction behavior rather than as direct measures of detection accuracy. Notably, the bright B16BL6 configuration achieved the highest inter-model consensus value, although the subsequent ground-truth evaluation showed substantially lower detection performance than the CycleGAN-translated configuration. This discrepancy shows that cross-model agreement can arise from correlated prediction patterns, including shared false detections or systematic over-detection, and may therefore be poorly aligned with true accuracy.

Consequently, $R_{\mathrm{cons}}$ is used only as a descriptive agreement measure. Similarly, the composite score represents a heuristic combination of confidence, inter-model agreement, and inverse detection density and should not be interpreted as a validated measure of detector performance. Comparative accuracy is established through the ground-truth evaluation presented in Section 3.4.

### 3.4. Ground-Truth Evaluation on the Annotated HeLa Subset

To provide direct quantitative validation on the target domain, we manually annotated a subset of 100 HeLa microscopy frames with bounding boxes for the Cell and Division classes. The subset consisted of 726 annotated instances, including 671 Cell instances and 55 Division instances. The annotations were used exclusively for the final evaluation and were not used in CycleGAN training, YOLOv8x training, checkpoint selection, or hyperparameter optimization. Therefore, the proposed unsupervised annotation transfer framework was preserved.

The three YOLOv8x configurations were evaluated on the same annotated subset of HeLa cells and with the same inference settings. Standard object detection metrics were calculated, including precision, recall, F1-score, mAP@0.50, and mAP@0.50:0.95. For all three models, validation was performed using identical settings: image size = 640, batch size = 8, confidence threshold = 0.001, non-maximum suppression (NMS) IoU threshold = 0.70, and maximum detections per image = 300. The low confidence threshold was used only for validation to construct complete precision–recall curves for average precision (AP) and mAP calculation and was not intended as a deployment-time prediction threshold.

As shown in Table 4, the detector trained on CycleGAN-translated B16BL6 → HeLa images achieved the highest performance across all the supervised metrics. It obtained a precision of 0.244, a recall of 0.353, an F1-score of 0.288, an mAP@0.50 of 0.186, and an mAP@0.50:0.95 of 0.055. In comparison, the bright B16BL6 baseline achieved a precision of 0.061, recall of 0.155, F1-score of 0.088, mAP@0.50 of 0.027, and mAP@0.50:0.95 of 0.010, while the dark B16BL6 baseline achieved a precision of 0.025, recall of 0.113, F1-score of 0.041, mAP@0.50 of 0.009, and mAP@0.50:0.95 of 0.004.

Training on CycleGAN-translated images improved mAP@0.50 by approximately 580%, F1-score by 229%, precision by 300%, and recall by 128% relative to the bright B16BL6 baseline. The results provide direct ground-truth evidence of improved detector performance in the HeLa target domain relative to source-only training.

Nevertheless, the absolute performance remains moderate, particularly under the stricter mAP@0.50:0.95 criterion. This indicates that, although CycleGAN-based annotation transfer reduces the B16BL6 → HeLa domain gap, residual differences in cell morphology, contrast distribution, imaging characteristics, and annotation distribution continue to affect target-domain detection. Therefore, the supervised HeLa evaluation should be interpreted as evidence of improved cross-domain generalization rather than as a fully supervised target-domain detection result.

Table 5 presents the class-level results and shows that the CycleGAN-translated configuration improves detection for both classes. This improvement was especially clear for the Cell class, where AP@0.50 increased from 0.044 for the bright B16BL6 baseline to 0.270 for CycleGAN B16BL6 → HeLa. The Division class remained more challenging due to the limited number of 55 ground-truth instances in the annotated HeLa subset and because mitotic morphology varies more strongly across imaging domains. However, the CycleGAN-trained model achieved the highest Division-class AP@0.50 and AP@0.50:0.95 among the compared configurations.

The newly introduced manually annotated HeLa subset provides direct quantitative evidence that the proposed annotation transfer framework improves target-domain detection performance relative to both baseline configurations. Nevertheless, the present evaluation remains restricted to a single source–target microscopy pair. Therefore, while the observed improvements demonstrate the effectiveness of the proposed framework under the investigated B16BL6 → HeLa domain shift, additional experiments involving different cell types, microscope systems, and acquisition protocols will be necessary to determine the extent to which these findings generalize to broader microscopy applications.

### 3.5. Annotation Geometry Preservation Results

Of the 1791 original–translated image pairs generated during CycleGAN inference, 1148 pairs (64.1%) satisfied the predefined RANSAC registration quality criteria, while the remaining 643 pairs (35.9%) were classified as registration-inconclusive due to their inability to establish reliable affine registration. The main causes of unsuccessful registration were insufficient feature correspondences, insufficient RANSAC inliers, or implausible affine transformations resulting from high repetition in the cellular structure and substantial variations in photometric appearance.

Conditional on successful registration, the estimated geometric transformations for the 1148 valid image pairs were close to the identity transformation. All the displacement, affine transformation, and transformed bounding-box IoU statistics reported below were calculated exclusively for this subset and should not be generalized to the complete translated dataset. The median displacement of RANSAC inlier keypoints was 1.18 pixels (IQR: 1.00–1.39 pixels), and the 95th percentile was 1.74 pixels, as summarized in Table 6. The median absolute affine translation was 1.54 pixels (interquartile range 1.00–2.13 pixels), the median absolute rotation deviation was $0.029^{\circ }$, and the median absolute scale deviation was 0.068%. Also, the median inlier displacement was less than two pixels for 98.34% of the valid registrations, and the affine translation estimate was less than five pixels for 98.52% of the valid registrations.

To assess the practical impact of these transformations on annotation validity, each original YOLO bounding box was transformed using the estimated affine transformation. For the 38,895 transformed annotations, the median IoU between each original bounding box and its transformed counterpart was 0.953 (IQR: 0.932–0.969), with a fifth percentile of 0.885. Moreover, 99.94% of the transformed bounding boxes retained an IoU greater than or equal to 0.50, 99.66% retained an IoU of at least 0.75, and 99.64% exhibited a bounding-box center displacement of no more than five pixels.

The supplementary structural analysis produced a median gradient-based SSIM of 0.556 and a median edge F1-score of 0.220. Since these measures are affected by the intended photometric modifications introduced by CycleGAN, they should be interpreted as complementary structural indicators rather than direct measures of geometry preservation.

**Figure 3:**
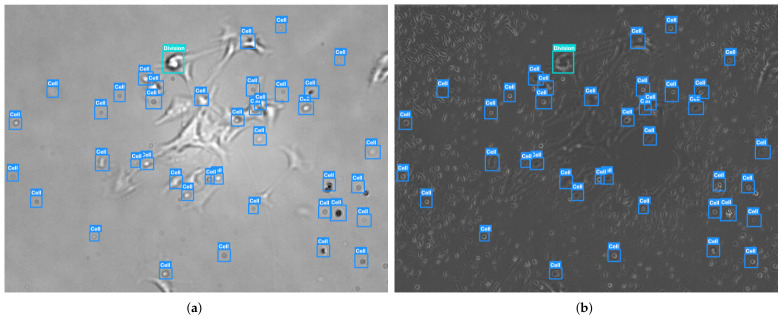
Qualitative illustration of annotation preservation during CycleGAN-based source-to-target translation. The identical source-domain bounding-box annotations are overlaid on (**a**) the original B16BL6 image and (**b**) its corresponding CycleGAN-translated B16BL6 → HeLa image. Blue boxes denote the Cell class, whereas cyan boxes denote the Division class. The comparison illustrates that the image appearance changes substantially while the transferred annotations remain visually associated with the corresponding cellular locations. This example provides qualitative support only; the quantitative geometry preservation findings are restricted to image pairs satisfying the predefined registration quality criteria.

To complement the quantitative registration-based analysis, Figure 3 presents an original B16BL6 image and its corresponding CycleGAN-translated image with the identical source-domain annotations overlaid on both panels. The bounding-box coordinates and class labels were not recalculated or predicted separately for the translated image; rather, the original annotations were directly reused. Therefore, the figure visually illustrates the central annotation transfer assumption investigated in this study.

Overall, the registration-based analysis supports approximate geometric preservation only for the 1148 original–translated image pairs (64.1%) that satisfied the predefined registration quality criteria; it does not establish geometry preservation for the complete translated dataset. The reported displacement, affine transformation, and transformed bounding-box metrics were calculated exclusively for these valid registrations. The remaining 643 pairs (35.9%) were registration-inconclusive because sufficiently reliable feature correspondences could not be established. Consequently, these cases provide neither evidence of preserved geometry nor evidence of geometric failure. The qualitative example in Figure 3 visually complements, but does not extend, this quantitative conclusion.

### 3.6. Sensitivity Analysis of Composite Score Weights

The composite reliability metric amalgamates various unsupervised proxy indicators to evaluate the quality of detection within the unlabeled target domain. To ascertain that the results presented are not contingent upon a particular selection of weights, a sensitivity analysis was conducted by recalculating the composite score across multiple representative weight configurations. In particular, five settings were evaluated: a default configuration $\left(w_{C},w_{R},w_{N}\right)=\left(0.5,0.3,0.2\right)$, an equal-weight configuration (0.33,0.33,0.34), a confidence-heavy configuration (0.6,0.2,0.2), a consensus-heavy configuration (0.3,0.5,0.2), and a count-regularized configuration (0.4,0.2,0.4), where $w_{C}$, $w_{R}$, and $w_{N}$ denote the weights assigned to normalized confidence, normalized inter-model consensus, and normalized inverse detection density, respectively. For each weighting configuration, the same normalized terms defined in Equation (7) were retained, and only their respective weights were changed.

Table 7 summarizes the tested weighting schemes, where, across all five settings, the model trained on CycleGAN-translated source images consistently achieved the highest reliability score on the HeLa target dataset with an average rank of 1.0 and no observed rank variability. On the other hand, the baseline models trained on bright and dark images demonstrated lower and less consistent ranks across the same configurations.

Across the five tested weighting schemes, the CycleGAN-translated configuration retained the highest composite score rank. This result indicates that the exploratory ranking is stable under the examined coefficient variations. However, the analysis does not validate the composite score as a measure of detection accuracy. Comparative performance is established primarily through the ground-truth evaluation reported in Section 3.4.

### 3.7. Qualitative Results

**Figure 4:**
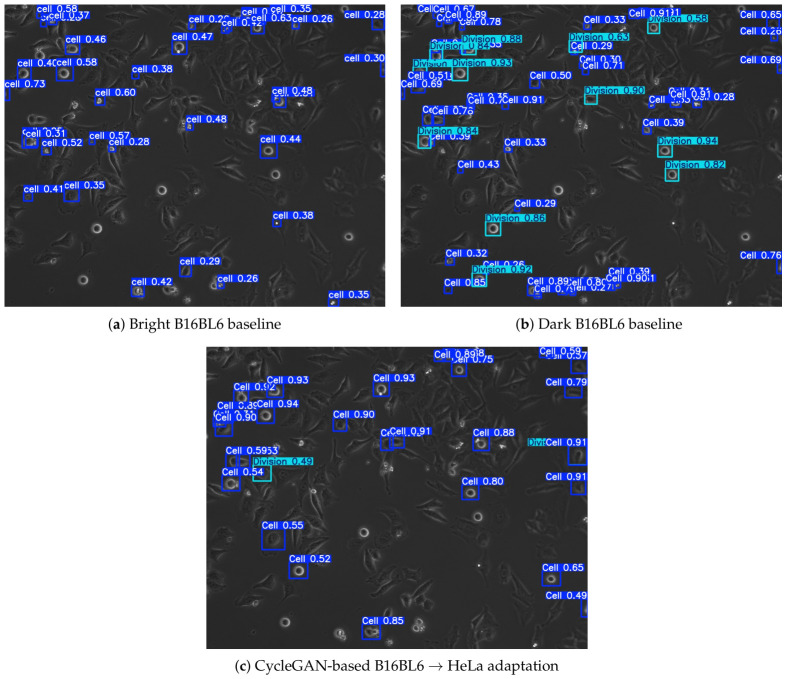
Qualitative comparison of YOLOv8x detection results on the same target-domain HeLa microscopy frame using three training configurations: (**a**) the original bright B16BL6 dataset, (**b**) the intensity-inverted dark B16BL6 dataset, and (**c**) the proposed CycleGAN-translated B16BL6 → HeLa dataset. Blue bounding boxes indicate predictions classified as Cell, whereas cyan bounding boxes indicate predictions classified as Division. The high-resolution images and enlarged two-column presentation improve the visibility of small cellular structures, bounding boxes, class labels, and confidence scores.

To support the analysis of the numerical proxy metric and to visually see how detection performance changes when the conditions change, we show comparisons for each of the three training setups using the same HeLa microscopy images. Figure 4 presents typical YOLOv8x detection outputs obtained from models trained using bright B16BL6 images, inverted (dark) B16BL6 images, and CycleGAN-translated B16BL6 → HeLa images.

First, the YOLOv8x model trained using bright B16BL6 images demonstrates substantial over-detection during testing on HeLa microscopy images. A large number of low-confidence bounding boxes appear in the background and long shapes, showing that the model is very sensitive to differences in lighting between the source and target images. This behavior aligns with the high detection density (85.7 detections per image) alongside the low mean confidence value (0.44), as reported in Table 3.

Second, the intensity inversion changed the number and confidence distribution of the predictions on the complete HeLa sequence. However, the ground-truth evaluation showed that the dark configuration performed worse than the bright baseline across all the supervised metrics. Therefore, the reduced prediction density should not be interpreted as reduced false-positive error because it may instead reflect missed detections. These results indicate that matching contrast polarity alone is insufficient to reproduce the more complex target-domain appearance.

Compared to the first two configurations, the CycleGAN-translated training model generates detections that are spatially aligned with the observed cell bodies while maintaining consistently higher confidence scores. Background-related spurious detections are largely eliminated, with the predicted number of bounding boxes matching more closely to the visible cellular density in the image. Overall, these observations qualitatively support the higher prediction confidence and improved ground-truth detection performance of the CycleGAN configuration. They should not be interpreted as validating inter-model consensus or the exploratory composite score as measures of accuracy.

In summary, the qualitative findings support the quantitative analysis and indicate that annotation-preserving image translation effectively reduces domain shift in phase-contrast microscopy, thereby improving the reliability and interpretability of detection outputs on unlabeled data from the target domain.

## 4. Discussion and Limitations

Despite the encouraging findings, several limitations should be acknowledged. First, direct target-domain validation was performed on a uniformly sampled and manually annotated subset of 100 HeLa frames, while the remainder of the target sequence remained unlabeled. Consequently, the standard object detection metrics characterize performance on this evaluation subset rather than on the complete HeLa domain. The full-sequence proxy indicators provide complementary descriptive information, but they do not replace ground-truth evaluation. Future work should expand the independently annotated HeLa subset and include a larger target-domain dataset that can be divided into separate training, validation, and test partitions. This would also enable comparison with a fully supervised HeLa baseline without reusing the current evaluation frames.

Second, the quality of the transferred annotations depends on the reliability of the source-domain labels. Although the B16BL6 pseudo-labels were verified by a biological expert and refined using spatiotemporal information, they were initially generated by a detector trained on 342 manually annotated frames. Residual errors, including missed objects, inaccurate localization, class-label confusion, or incomplete annotation in densely populated regions, may therefore remain. Because a separate subset containing exclusively independent manual B16BL6 annotations was not retained, a retrospective comparison between purely manual and model-assisted source annotations could not be performed. Such source annotation errors do not affect CycleGAN optimization directly because the translation model does not use bounding-box labels, but they may propagate to the translated training dataset and influence detector performance. Future work should therefore investigate annotation-quality filtering and robustness to controlled source-label perturbations.

Third, the experimental validation is restricted to one source–target pair, namely B16BL6 and HeLa phase-contrast microscopy. Although this scenario represents a realistic cross-laboratory domain shift, it does not capture the full diversity of cell types, morphologies, microscope systems, resolutions, contrast conditions, and acquisition protocols encountered in biomedical imaging. Extension to additional datasets is also complicated by differences in annotation formats, class definitions, and biological targets. Broader evaluation will therefore require annotation harmonization and, in some cases, additional expert labeling before comparable object detection experiments can be conducted.

Fourth, the comparative evaluation was intentionally limited to the original bright B16BL6 baseline, the intensity-inverted dark B16BL6 baseline, and the CycleGAN-translated configuration. These controlled settings were selected to isolate the effect of the training-image domain while keeping the detector architecture and training settings fixed. However, the present study does not provide a comprehensive benchmark against recent unsupervised domain adaptation methods such as self-training, confidence-based pseudo-label refinement, teacher–student learning, feature-level alignment, or task-coupled adversarial optimization. Similarly, all the detector experiments were conducted using YOLOv8x. Although this architecture was selected based on its strong performance in our previous B16BL6 comparison, the present findings do not establish that the observed improvement is independent of detector architecture or model size. Future studies should therefore compare the framework with representative UDA approaches and evaluate smaller detector variants such as YOLOv8s and YOLOv8m.

Fifth, the geometry preservation findings apply only to the 64.1% of original–translated image pairs for which reliable RANSAC-based registration was obtained. For these valid registrations, the affine transformation and transformed bounding-box analyses indicated limited measurable spatial change and substantial annotation overlap. The remaining 35.9% of pairs were registration-inconclusive because repetitive cellular morphology and strong photometric variation prevented sufficiently reliable feature correspondences from being established. These cases cannot be interpreted as either geometrically preserved or geometrically distorted. The qualitative comparison of identical annotations overlaid on original and translated images provides additional visual support, but it does not extend the quantitative conclusion beyond the validly registered subset. Future work should incorporate instance-level validation based on independently annotated translated images or stable biological landmarks.

Finally, the supervised target-domain results show that substantial residual domain differences remain, particularly under stricter localization criteria and for the underrepresented Division class. This indicates that appearance translation alone cannot fully address differences in cellular morphology, class distribution, imaging characteristics, and annotation patterns. Combining image translation with feature-level adaptation, uncertainty-aware pseudo-label refinement, semi-supervised learning, or target-domain fine-tuning may further improve performance while retaining the annotation efficiency benefits of the proposed framework.

Overall, the proposed framework provides a practical annotation transfer strategy for the investigated B16BL6 → HeLa phase-contrast microscopy domain shift. The findings support the usefulness of source-to-target CycleGAN translation for improving cross-domain detector training relative to the evaluated baselines, while the limitations above define the scope within which the results should be interpreted. Broader claims of generalization will require evaluation across additional microscopy datasets, detector architectures, and domain adaptation methods.

## 5. Conclusions

This study presented a detection-oriented annotation transfer framework for cross-domain phase-contrast microscopy based on CycleGAN image translation. Instead of adapting the target domain to the source domain, annotated B16BL6 microscopy images were translated into the visual appearance of the HeLa domain while enabling reuse of their original bounding-box annotations under an empirically evaluated spatial-correspondence assumption, enabling YOLOv8x training without manual annotation of the complete target dataset.

To assess the proposed framework, we combined two complementary evaluation strategies. We computed standard object detection metrics, including precision, recall, F1-score, and mAP, on a manually annotated subset of 100 HeLa images, while the proposed unsupervised proxy metrics were used to evaluate large-scale detection performance on the remaining unlabeled target-domain images. The quantitative evaluation demonstrated that the CycleGAN-translated detector outperformed both the bright and dark baseline configurations on all the metrics, demonstrating that source-to-target translation with reused source annotations improved detector performance under the tested B16BL6 → HeLa domain shift. The supervised and full-sequence proxy analyses characterize different aspects of target-domain behavior. The ground-truth evaluation provides the primary evidence of detection performance, whereas the proxy indicators provide only complementary descriptive information for the remaining unlabeled sequence.

Although the proposed framework reduced the appearance discrepancy between the B16BL6 and HeLa domains relative to the evaluated baselines, the present study constitutes a controlled validation on a single source–target microscopy pair. Thus, the results should be interpreted as evidence of improved cross-domain adaptation in the B16BL6 → HeLa scenario and not as proof of universal generalization over microscopy domains. Future work will investigate additional microscopy datasets and source–target combinations, evaluate smaller detector variants, and benchmark the framework against representative unsupervised domain adaptation methods under standardized experimental conditions.

## Figures and Tables

**Table 1:** Cycle-Consistent Generative Adversarial Network (CycleGAN) training configuration used to generate the B16BL6 → HeLa translated dataset.

Parameter	Setting
Generator architecture	ResNet generator with nine residual blocks
Discriminator architecture	70×70 PatchGAN
Normalization and padding	Instance normalization and reflection padding
Adversarial objective	Least-squares GAN (LSGAN)
Cycle-consistency weight	$\lambda_{\mathrm{cyc}}=10$
Identity-loss scaling factor	$\lambda_{\mathrm{identity}}=0.5$
Optimizer	Adam (learning rate $=2\times 10^{-4}$, $\beta_{1}=0.5$)
Learning-rate schedule	Constant for 100 epochs, followed by linear decay over 100 epochs
Scheduled training duration	200 epochs
Batch size	4
Training preprocessing	Resize to 512×512, followed by random crop to 256×256
Training flip augmentation	Disabled
Translated-dataset inference	Original image resolution without cropping or horizontal flipping
Checkpoint used for dataset generation	Epoch 122
Checkpoint-selection criterion	Pre-evaluation visual assessment of translation stability and structural artifacts

**Table 2:** YOLOv8x training configuration used for the bright B16BL6, dark B16BL6, and CycleGAN-translated B16BL6 → HeLa detector configurations.

Parameter	Setting
Model initialization	COCO-pretrained YOLOv8x weights
Training epochs	100
Input resolution	640×640 pixels
Batch size	16
Remaining training parameters	Unmodified Ultralytics YOLOv8x defaults

**Table 3:** Descriptive full-sequence proxy statistics measured on the HeLa target dataset. Values are reported as mean ± standard deviation (SD) where applicable. Consensus denotes cross-model spatial agreement only, and the composite score is an exploratory weighted summary of the selected indicators; neither should be interpreted as a calibrated measure of detection accuracy or reliability.

Model	Mean Confidence	Median Confidence	Detections/ Image	Consensus	Box Area ($\times 10^{-3}$)	Composite
Bright B16BL6 (Baseline)	0.439 ± 0.18	0.41	85.7 ± 43.9	0.852	0.7 ± 0.4	0.726
Dark B16BL6 (Inverted)	0.577 ± 0.15	0.59	46.9 ± 12.4	0.485	0.8 ± 0.3	0.786
CycleGAN (B16BL6 → HeLa)	**0.692 ** ± 0.12	**0.77**	**46.4** ± 17.9	**0.662**	**2.0** ± 0.6	**0.933**

**Table 4:** Ground-truth evaluation on the manually annotated 100-frame HeLa target-domain subset. The best result in each column is shown in bold.

Training Configuration	Precision	Recall	F1-Score	mAP@0.50	mAP@0.50:0.95
Bright B16BL6	0.061	0.155	0.088	0.027	0.010
Dark B16BL6	0.025	0.113	0.041	0.009	0.004
CycleGAN B16BL6 → HeLa	**0.244**	**0.353**	**0.288**	**0.186**	**0.055**

**Table 5:** Class-level performance on the annotated HeLa subset.

Model	Class	Precision	Recall	F1-Score	AP@0.50	AP@0.50:0.95	Ground-Truth Instances
Bright B16BL6	Cell	0.072	0.219	0.108	0.044	0.015	671
Bright B16BL6	Division	0.050	0.091	0.065	0.011	0.006	55
Dark B16BL6	Cell	0.035	0.134	0.055	0.016	0.006	671
Dark B16BL6	Division	0.015	0.091	0.026	0.003	0.002	55
CycleGAN B16BL6 → HeLa	Cell	**0.307**	**0.505**	**0.382**	**0.270**	**0.067**	671
CycleGAN B16BL6 → HeLa	Division	**0.180**	**0.200**	**0.190**	**0.102**	**0.043**	55

**Table 6:** Annotation geometry preservation results conditional on successful registration. The reported statistics were calculated for the 1148 image pairs (64.1% of the 1791 available original–translated pairs) that satisfied the predefined RANSAC registration quality criteria. The remaining 643 pairs (35.9%) were registration-inconclusive and were excluded from all displacement, affine transformation, and transformed bounding-box IoU estimates.

Metric	Median [Q1, Q3]	P5	P95
RANSAC inlier displacement (pixels)	1.183 [1.003, 1.391]	0.744	1.744
Inlier keypoints within 2 pixels	0.833 [0.750, 0.900]	0.583	1.000
Inlier keypoints within 5 pixels	1.000 [1.000, 1.000]	1.000	1.000
Absolute affine translation (pixels)	1.535 [1.000, 2.130]	0.415	3.721
Absolute affine rotation (°)	0.029 [0.015, 0.054]	0.002	0.118
Absolute scale deviation (%)	0.068 [0.032, 0.117]	0.006	0.232
Original–transformed bounding-box IoU	0.953 [0.932, 0.969]	0.885	0.986

**Table 7:** Ranking stability across different composite score weight configurations.

Model	Best Count	Average Rank	Minimum	Maximum
CycleGAN B16 → HeLa	5	1.0	1	1
Dark B16BL6	0	2.2	2	3
Bright B16BL6	0	2.8	2	3

## Data Availability

The B16BL6 microscopy dataset and its YOLO-format annotations are publicly available through the Open Science Framework at https://doi.org/10.17605/OSF.IO/5J276. The HeLa microscopy dataset and the manually prepared HeLa evaluation annotations are not publicly available.
